# Antibiotics needed to treat multidrug-resistant infections in neonates 

**DOI:** 10.2471/BLT.22.288623

**Published:** 2022-10-03

**Authors:** Phoebe CM Williams, Shamim A Qazi, Ramesh Agarwal, Sithembiso Velaphi, Julia A Bielicki, Sumathi Nambiar, Carlo Giaquinto, John Bradley, Gary J Noel, Sally Ellis, Seamus O’Brien, Manica Balasegaram, Michael Sharland

**Affiliations:** a School of Public Health, Faculty of Medicine, Edward Ford Building, The University of Sydney, Camperdown, NSW, 2006, Australia.; b Geneva, Switzerland.; c Department of Paediatrics, All India Institute of Medical Sciences, New Delhi, India.; d Department of Paediatrics, Chris Hani Baragwanath Academic Hospital, Johannesburg, South Africa.; e Institute of Infection and Immunity, University of London, London, England.; f Johnson & Johnson, Rockville, United States of America (USA).; g Department of Women and Children’s Health, University of Padua, Padua, Italy.; h Department of Pediatric Infectious Diseases, University of California San Diego School of Medicine, San Diego, USA.; i Institute for Advanced Clinical Trials for Children, Weill Cornell Medical College, Rockville, USA.; j Global Antibiotic Research and Development Project, Geneva, Switzerland.

## Abstract

Infections remain a leading cause of death in neonates. The sparse antibiotic development pipeline and challenges in conducting neonatal research have resulted in few effective antibiotics being adequately studied to treat multidrug-resistant (MDR) infections in neonates, despite the increasing global mortality burden caused by antimicrobial resistance. Of 40 antibiotics approved for use in adults since 2000, only four have included dosing information for neonates in their labelling. Currently, 43 adult antibiotic clinical trials are recruiting patients, compared with only six trials recruiting neonates. We review the World Health Organization (WHO) priority pathogens list relevant to neonatal sepsis and propose a WHO multiexpert stakeholder meeting to promote the development of a neonatal priority antibiotic development list. The goal is to develop international, interdisciplinary consensus for an accelerated neonatal antibiotic development programme. This programme would enable focused research on identified priority antibiotics for neonates to reduce the excess morbidity and mortality caused by MDR infections in this vulnerable population.

## Résumé

Les infections demeurent l'une des principales causes de décès chez les nouveau-nés. Les rares projets de développement d'antibiotiques et les défis posés par la recherche néonatale ont entraîné une pénurie d'antibiotiques efficaces spécialement étudiés pour traiter les infections multirésistantes (MR) chez les nouveau-nés, en dépit d'une mortalité galopante due à une résistance accrue aux antimicrobiens. Sur 40 antibiotiques autorisés pour les adultes depuis 2000, quatre à peine sont munis d'un étiquetage indiquant la posologie adaptée aux nouveau-nés. Actuellement, 43 essais cliniques portant sur des antibiotiques recrutent des patients du côté des adultes, contre six seulement du côté des nouveau-nés. Dans le présent document, nous passons en revue la liste prioritaire d'agents pathogènes établie par l'Organisation mondiale de la Santé (OMS) pour soigner la septicémie néonatale et proposons de réunir, sous l'égide de l'OMS, des parties prenantes issues de plusieurs domaines d'expertise afin de promouvoir la création d'une liste prioritaire de développement d'antibiotiques destinés aux nouveau-nés. Objectif: parvenir à un consensus international et interdisciplinaire visant à accélérer le programme de mise au point d'antibiotiques à usage néonatal. Ce programme permettrait d'orienter les recherches vers des antibiotiques identifiés comme prioritaires pour les nouveau-nés, en vue de faire baisser les taux de morbidité et de mortalité excessifs qu'engendrent les infections MR au sein de cette population vulnérable.

## Resumen

Las infecciones siguen siendo una de las principales causas de muerte en los recién nacidos. Debido al escaso desarrollo de los antibióticos y a las dificultades para llevar a cabo la investigación neonatal, son pocos los antibióticos eficaces que se estudian de manera adecuada para tratar las infecciones multirresistentes (MR) en los recién nacidos, a pesar de la creciente carga de mortalidad mundial causada por la resistencia a los antimicrobianos. De los 40 antibióticos aprobados para su uso en adultos desde el 2000, solo cuatro han incluido información sobre la dosis para recién nacidos en su etiquetado. En la actualidad, 43 ensayos clínicos con antibióticos para adultos están reclutando pacientes, en comparación con solo seis ensayos que reclutan recién nacidos. Se revisa la lista de patógenos prioritarios de la Organización Mundial de la Salud (OMS) relevantes para la sepsis neonatal y se propone una reunión de la OMS con múltiples expertos para promover el desarrollo de una lista de antibióticos prioritarios para los recién nacidos. El objetivo es desarrollar un consenso internacional e interdisciplinario para establecer un programa acelerado de desarrollo de antibióticos neonatales. Este programa permitiría centrar la investigación en los antibióticos prioritarios identificados para los recién nacidos con el fin de reducir el exceso de morbilidad y mortalidad causado por las infecciones MR en esta población vulnerable.

## ملخص

تظل العدوى سببًا رئيسيًا للوفيات بين الأطفال حديثي الولادة. أدت المضادات الحيوية المتفرقة الجاري تطويرها والتحديات التي تواجه إجراء أبحاث على الأطفال حديثي الولادة إلى دراسة عدد قليل وحسب من المضادات الحيوية الفعالة بشكل كافٍ لعلاج العدوى المقاومة للأدوية المتعددة (MDR) لدى المواليد، وذلك على الرغم من زيادة عبء الوفيات العالمي الناجم عن مقاومة مضادات الميكروبات. من بين 40 مضادًا حيويًا تم اعتمادها واستخدمها البالغون منذ عام 2000، تتضمن أربعة منها فقط معلومات عن الجرعة المناسبة للأطفال حديثي الولادة في التوسيم الخاص بها. وفي الوقت الحالي، تُجرى 43 تحربةٓ سريريةٓ لدراسة المضادات الحيوية على مرضى بالغين، مقارنةً بست (6) تجارب فقط تستعين بأطفال حديثي الولادة لإجراء التجارب. نحن نراجع قائمة العوامل الممرضة البكتيرية ذات الأولوية التي أعدتها منظمة الصحة العالمية (WHO) والمرتبطة بإنتان حديثي الولادة ونقترح عقد اجتماع لمنظمة الصحة العالمية يضم أصحاب المصلحة المعنيين من ذوي الخبرات المتنوعة بغية تعزيز الجهود الرامية لإعداد قائمة لتطوير المضادات الحيوية ذات الأولوية للأطفال حديثي الولادة. الهدف من هذا الأمر هو تشكيل توافق آراء دولي يشمل تخصصات متعددة بشأن الإسراع بوتيرة برنامج تطوير المضادات الحيوية الخاصة بالأطفال حديثي الولادة. سيتيح هذا البرنامج الفرصة للتركيز على الأبحاث التي تتم على المضادات الحيوية ذات الأولوية والخاصة بالأطفال حديثي الولادة بغرض تقليل ارتفاع معدلات الإصابات والوفيات الناتجة عن العدوى المقاومة للأدوية المتعددة في هذه الفئة الضعيفة من السكان.

## 摘要

感染仍然是导致新生儿死亡的主要原因。尽管抗菌素耐药性导致全球死亡率负担不断增加,但匮乏的抗生素开发渠道和进行新生儿研究方面的挑战导致就治疗新生儿的多重耐药性 (MDR) 感染进行充分研究的有效抗生素数量非常有限。自 2000 年来批准用于成人的 40 种抗生素中,只有 4 种药物在其标签中包含了新生儿用药剂量信息。目前,有 43 项成人抗生素临床试验正在招募患者,而仅有 6 项试验在招募新生儿。我们审查了世卫组织 (WHO) 与新生儿败血症相关的优先病原体清单,并提议召开世卫组织多专家利益相关者会议,以促进新生儿首选抗生素开发清单的制定。目标是达成国际跨学科共识,以加速新生儿抗生素开发计划的制定。该计划将使研究专注于确定的新生儿首选抗生素上,以便降低这一易受感染人群中由多重耐药性感染引起的过高发病率和死亡率。

## Резюме

Инфекции остаются основной причиной смерти новорожденных. Небольшое количество разрабатываемых антибиотиков и трудности при проведении неонатальных исследований привели к тому, что сейчас должным образом изучают лишь несколько эффективных антибиотиков для лечения инфекций со множественной лекарственной устойчивостью (МЛУ) у новорожденных, несмотря на растущее глобальное бремя смертности, вызванное устойчивостью к противомикробным препаратам. Из 40 антибиотиков, одобренных для применения у взрослых с 2000 года, только в четырех инструкциях по медицинскому применению препарата содержится информация о дозировке для новорожденных. В настоящее время проводятся 43 клинических испытания антибиотиков с участием взрослых пациентов по сравнению лишь с шестью испытаниями, в которых участвуют новорожденные. Авторы рассматривают список приоритетных патогенов Всемирной организации здравоохранения (ВОЗ), имеющих отношение к неонатальному сепсису, а также предлагают провести совещание заинтересованных сторон с участием многих экспертов ВОЗ для содействия разработке списка приоритетных антибиотиков для новорожденных. Цель состоит в том, чтобы достичь международного междисциплинарного консенсуса в отношении ускоренной программы разработки антибиотиков для новорожденных. Эта программа позволит проводить целенаправленные исследования по установленным приоритетным антибиотикам для новорожденных, чтобы снизить избыточную заболеваемость и смертность, вызванные МЛУ-инфекциями, внутри этой уязвимой группы населения.

## Introduction

Despite a significant decline in deaths in children younger than 5 years globally in the past three decades, preventable and treatable infectious diseases remain the leading cause of death in this age group. This problem is further exacerbated by the global rise of antimicrobial resistance.^1^^,^^2^ Neonates are particularly vulnerable to systemic infections caused by multidrug-resistant (MDR) microorganisms, because of their immature immune systems and increased risk of hospital-acquired infections – particularly where hospital stays are prolonged because of prematurity or congenital anomalies.^3^

Systemic infections are one of the main causes of about 2.3 million neonatal deaths occurring each year globally. An estimated 3 million cases of neonatal sepsis occur each year, resulting in up to 570 000 deaths attributable to sepsis; many of these deaths are because of the lack of efficacy of available antibiotics.^4^

As an increasing proportion of births occurs in health-care settings worldwide, early colonization with MDR pathogens is becoming more common, and MDR gram-negative pathogens are now responsible for a substantial burden of severe disease caused by neonatal sepsis.^2^^,^^5^ In India alone, an estimated 60 000 neonates die each year from sepsis caused by bacteria resistant to first-line antibiotics,^6^ and gram-negative infections now account for more than three quarters of all neonatal culture-positive deaths.^7^

**Fig. 1:**
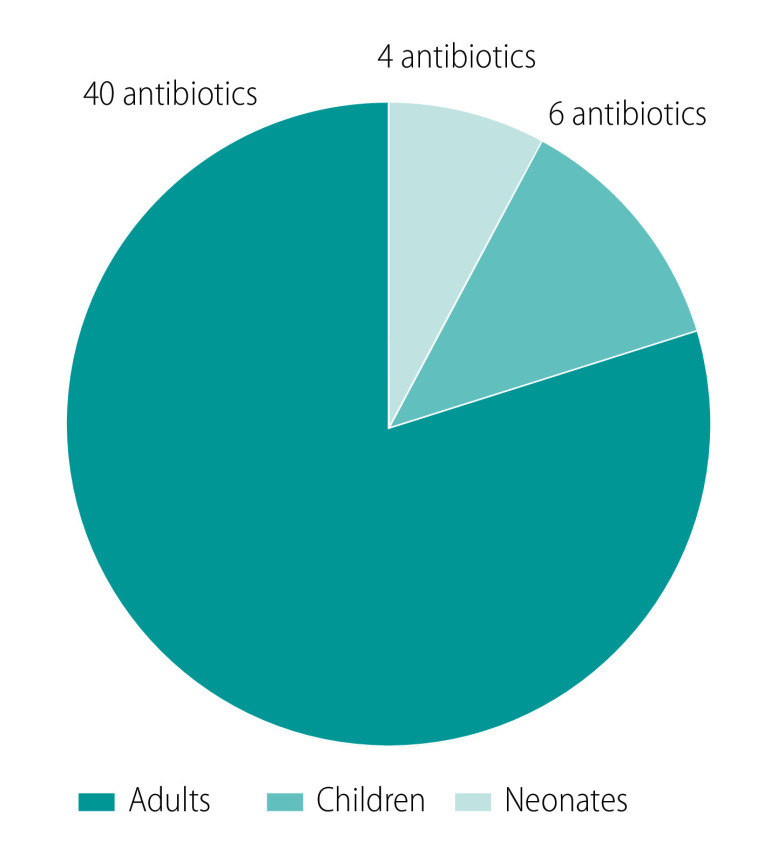
Number of antibiotics approved for use since 2000, by age cohort, 2022

Note: We systematically collated data from the ClinicalTrials.gov database, Food and Drug Administration, European Medicines Agency, PEW Charitable Trusts,^8^ World Health Organization^9^ and Butler et al.^10^^,^^11^

**Fig. 2:**
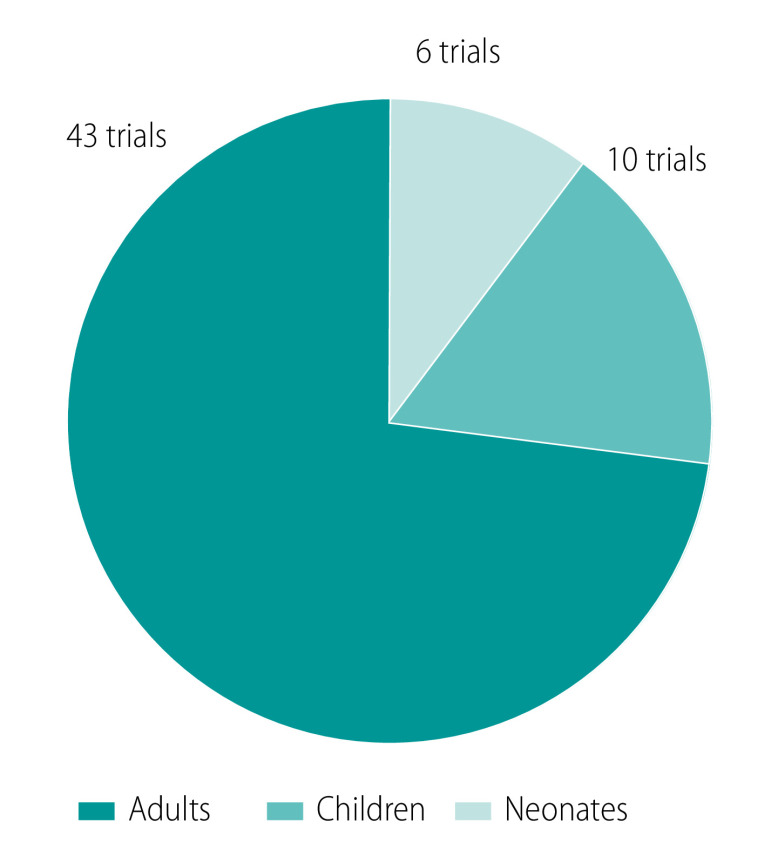
Number of antibiotic clinical trials recruiting patients, by age cohort, 2022

Note: We systematically collated data from the ClinicalTrials.gov database, Food and Drug Administration, European Medicines Agency, PEW Charitable Trusts,^8^ World Health Organization^9^ and Butler et al.^10^^,^^11^

Ensuring access to effective antibiotics in the context of increasingly high antimicrobial resistance rates is of critical importance. However, the current drug development and regulatory frameworks result in limited and delayed access to approved drugs (with labelling guidance) for all children, and in particular, neonates (Fig. 1).^12^ Despite their high rates of infections, neonates have seldom been identified as a high-priority population for inclusion in clinical research programmes because of the ethical, logistical, regulatory and technical challenges inherent to conducting trials in this age group.^12^^,^^13^ In the context of a sparse global antibiotic development landscape,^8^ far fewer trials investigating new antibiotics are currently being conducted in neonates than in adults: six neonatal trials compared with 43 adult trials (Fig. 2).^9^ Furthermore, the current delay in neonatal access to new agents is substantial; most of the antibiotics approved for use in adults in the past two decades are yet to be labelled for use in neonates, or were labelled more than 10 years after their approval for use in adults.^12^^,^^14^

This lack of research priority is particularly concerning given the extensive use of antibiotics among neonates. On any given day, up to 40% of infants admitted to a neonatal intensive care unit are prescribed antibiotics, with an estimated 90% exposed to antibiotic medications over the duration of their stay in the intensive care unit.^15^ Many of these antibiotics are prescribed off-label because of the perceived or documented need for empirical or targeted therapy of MDR pathogens. Such prescribing risks reducing efficacy or increasing toxicity because of under- or over-dosing; it also increases the potential for antimicrobial resistance selection pressure because of suboptimal dosing.

To enable evidence-based treatment guidance for MDR sepsis in neonates, both preclinical and clinical studies are urgently needed to define pharmacokinetic and pharmacodynamic parameters, as well as safety and efficacy data. We therefore aim to explore potential strategies to optimize the development of antibiotics against the most common pathogens causing neonatal sepsis globally, by proposing the development of a list of priority antibiotics for use in neonates. This list would define new antibiotics, which may already be approved for adults but not yet for neonates, that require expedited confirmation of their efficacy against the most common MDR pathogens driving high rates of neonatal mortality. The overarching goal is to develop a multidisciplinary consensus approach for a global accelerated clinical development programme. This programme will enable streamlined approval of antibiotics for use in neonates and enhance collaboration between resource-rich and resource-constrained countries in the face of increasing mortality caused by antimicrobial resistance.

## Unique pathophysiology of neonatal sepsis

Undertaking drug trials in neonates is challenging. Neonates are a heterogeneous population, with significant variation in postnatal and gestational age and weight. Within the short neonatal period of 4 weeks, important changes occur in the functional maturation of different organ systems. These factors can result in rapidly changing drug absorption, distribution, metabolism and excretion. Furthermore, variability in antibiotic exposure is often complicated by organ dysfunction and limited drug clearance because of comorbid conditions.

Consequently, high-quality pharmacokinetic and pharmacodynamic knowledge is lacking for many drugs used in neonates, which has resulted in considerable variation in dosing guidelines across different formularies.^16^ In addition, combination antibiotic therapy is often prescribed in neonates, which may complicate estimates of optimal pharmacokinetic and pharmacodynamic parameters. Another challenge lies in the definition and diagnosis of neonatal sepsis, which is the most common indication for antibiotic prescription in this age group.

The variable clinical manifestation of neonatal sepsis includes a broad spectrum of illness, and its diagnosis is reliant on combinations of nonspecific clinical symptoms and signs that may be indistinguishable from other minor or life-threatening conditions, including hypoxic complications from a difficult delivery. Thus, definitions of neonatal sepsis vary, resulting in the inclusion of non-standardized populations within clinical trials.^17^ Further challenges include the high rates of culture-negative sepsis evident in neonates – influenced by exposure to intrapartum and postnatal antibiotics – the difficulty of obtaining adequate neonatal blood culture volumes and limited microbiological capacity in many health-care settings.^18^^,^^19^

In adults, sepsis is regarded as predominantly occurring secondary to a primary infection source. Regulatory guidance on new antibiotic licensing is thus based generally on the underlying focal infection, such as complicated urinary tract infections. Yet unlike sepsis in adults, bacterial neonatal sepsis is usually diagnosed when the organism is isolated from the bloodstream, without any identifiable focal infection source. Even more frequently, diagnosis is based on clinical manifestations alone without a positive blood culture. Lack of this information limits confidence in extrapolating the efficacy of new antibiotics – established mainly by studying adults with focal infections – to neonates with sepsis.

Neonates are also more likely to experience central nervous system infections than other age groups because of their immature immune system and more permeable blood–brain barrier.^14^ Understanding a drug’s penetration into the central nervous system is therefore an important consideration in treating neonates.

Improved antimicrobial pharmacokinetic and pharmacodynamic data are therefore urgently needed for the neonatal population, but several barriers exist to conducting trials to ascertain these data. These complexities include the ethical and practical considerations of obtaining adequate blood samples and difficulties in defining the optimal probability of target attainment in the context of the unique and rapidly changing pathophysiology in neonates.^20^

## Neonatal antibiotic development

Given the challenges outlined, it is not surprising that the current landscape of neonatal labelling for newly approved antibiotics is very limited. Indeed, of the 40 antibiotics approved in adults since 2000 (list of these antibiotics available in the data repository),^21^ only four (linezolid, dalbavancin, ceftaroline fosamil and ceftolozane + tazobactam) include dosing information for neonates in their label (Fig. 1). No licensed antibiotic regimen to treat carbapenem-resistant infections in neonates currently exists apart from polymyxin B, which was licensed by the US Food and Drug Administration over five decades ago. Even so, few pharmacokinetic and safety data have been published to confirm polymyxin B’s role in neonatal sepsis.^22^

Currently, paediatric drug development is largely overseen by the US Food and Drug Administration and the European Medicines Agency, with significant variation in their legislative processes for antibiotic approval. The Food and Drug Administration mandates paediatric studies for drugs approved in adults with *de novo* efficacy and safety data generated through randomized standard-of-care comparator trial designs, often with a 4:1 randomization between the new agent and a mixed standard-of-care comparator.^12^ In contrast, the European Union’s regulatory approval process does not generally require *de novo* safety data from randomized comparative trials in children; nevertheless, about two thirds of paediatric drug development trials still fail to reach completion.^23^

While there are differences in their approaches to safety data for new antibiotics, both the US Food and Drug Administration and the European Medicines Agency accept extrapolation for most indications. Promisingly, the most recent Food and Drug Administration guidance for the development of anti-infective drug products advocates enhancing trials in children by promoting the enrolment of adolescents (≥ 12 years) in phase III adult trials, and ensuring sponsors consider paediatric study plans promptly at the completion of phase II trials.^24^ These recommendations also promote parallel, rather than sequential, enrolment of paediatric age cohorts to facilitate the acquisition of trial data, and some recent studies are now including collection of neonatal pharmacokinetic data in their paediatric programme. However, for other medicines, neonates continue to be recruited in separate studies only after other paediatric cohorts have completed enrolment, and separate recruitment of various gestational age and weight brackets continues to be recommended.^24^^,^^25^

Recognizing these difficulties, regulators may take a more flexible approach to drug approvals in neonates, as exemplified by the streamlined and pragmatic approach recently used to provide dosing guidance for the antifungal micafungin in the treatment of neonatal candidiasis. Micafungin’s limited efficacy and safety data were considered alongside non-clinical data after the early termination, because of unsuccessful recruitment, of an attempted phase III trial, to provide dosing information on the use of micafungin in proven candidaemia in neonates and young infants. This dosing information was accompanied by a clear explanation of the areas of remaining uncertainty. This example illustrates a flexible approach by which data from multiple sources can be collated to enable licensing of antibiotics for neonates with difficult-to-treat infections.

## Enabling access

### Identifying the key pathogens

In 2017, the World Health Organization (WHO) published a list of priority pathogens to focus research and development efforts on important causes of morbidity and mortality worldwide due to antimicrobial resistance.^26^ Although this initiative has been successful in focusing early drug development, it has not been adapted for at-risk populations, including neonates. We therefore re-evaluated the WHO priority pathogen list to focus on the key MDR pathogens causing neonatal sepsis globally (Box 1).^18^^,^^26^^,^^27^^,^^32^ We were guided by recent epidemiological studies that have noted antimicrobial resistance disproportionately affects neonates in low- and middle-income countries, with rates of MDR gram-negative infections exceeding 80% of causative pathogens in some clinical settings (Table 1).^18^^,^^28^

Box 1 **High-burden pathogens responsible for most excess morbidity and mortality in neonates due to multidrug-resistant infections, 2022**

Extended-spectrum β-lactamase-producing *Klebsiella *spp*.*

Extended-spectrum β-lactamase-producing *Escherichia coli*

Carbapenemase-producing *Klebsiella* spp. (particularly class B metallo-β-lactamase producers)

Carbapenemase-producing *E. coli* (particularly class B metallo-β-lactamase producers)

Carbapenem-resistant *Acinetobacter baumannii* complex

Carbapenemase-producing *Serratia marcescens*

Carbapenemase-producing *Enterobacter cloacae*

Carbapenem-resistant *Pseudomonas aeruginosa*

Methicillin-resistant *Staphylococcus aureus*

Vancomycin-resistant *S. aureus*

Vancomycin-resistant *Enterococcus faecium*

Note: Adapted from the World Health Organization list of priority pathogens,^26^ based on recent studies. ^4^^,^^18^^,^^27^^–^^31^

**Table 1:** Recent large-scale, multicentre observational studies of the pathogens causing neonatal sepsis and their findings

Author, year	Country(ies)	Setting	Population	Dates of recruitment	Incidence of culture-positive sepsis, %	Most frequent pathogen causing neonatal sepsis (from neonates with culture-positive sepsis)	Proportion that were MDR or resistance to key antibiotics
DeNIS collaboration, 2016^30^	India	Three urban tertiary hospitals	• 13 530 neonates admitted to intensive care units • 840 developed culture-positive sepsis • Followed daily until discharge or death	2011–2014	6.2% (840/13 530)	*Acinetobacter* spp. (22.1%; 222/1005)	81.5% (181/222)
						*Klebsiella* spp. (16.8%;169/1005)	53.8% (91/169)
						*Escherichia coli* (13.6%; 137/1005)	38.0% (52/137)
						*Staphylococcus aureus* (12.1%; 122/1005)	37.7% (43/114) of *S. aureus* isolates resistant to methicillin
Saha et al. (The ANISA Study), 2018^29^	Bangladesh, India, Pakistan	Community settings across five sites	• 63 114 infants visited at home by community health workers up to 10 times between ages 0 and 59 days • 6022 identified as having possible serious bacterial infections • 4859 had blood taken for culture	2011–2014	2.7% (132/4859), excluding contaminants; incidence of culture-confirmed infection = 1.6 per 1 000 live births	• *E. coli* (20.6%; 21/102) • *Klebsiella* spp. (16.7%; 17/102) • *S. aureus* (11.8%; 12/102) • Group A *streptococcus* (10.8%; 11/102)	• 25.0% of all 50 tested pathogenic gram-negative isolates not susceptible to penicillin, ampicillin or gentamicin • Incidence of gram-negative organisms higher among hospital-born than community-born infants (1.3/1000 live births vs 0.7/1000 live births)
Sands et al. (BARNARDS network), 2021^18^	Bangladesh, Ethiopia, India, Nigeria, Pakistan, Rwanda, South Africa	Network of 12 urban hospitals	• 36 285 neonates prospectively recruited in the peripartum period • 2483 developed culture-confirmed sepsis • Followed up until day 60, study withdrawal or death	2015–2017	6.8% (2 483/36 285)	• *K. pneumoniae* (10.4%; 258/2483) • *Serratia marcescens* (6.1%; 151/2483) • *K. michiganesis* (4.7%; 117/2483) • *E. coli* (3.0%; 75/2483) • *Enterobacter cloacae* complex (3.2%; 80/2482) • Gram-positive bacteria constituted 48.3% (1266/2620)	• Of 885 gram-negative pathogens, high levels of resistance found to ampicillin (95.0%), cefotaxime (83.0%), ceftriaxone (80.0%), meropenem (87.0%) and tigecycline (85.0%) • 67.0% (597/885) of gram-negative isolates resistant to at least one β-lactam and one aminoglycoside • Very high rates of class B and class D carbapenemases across gram-negative species • All isolated pathogens resistant to multiple antibiotic classes, including classes recommended as empirical treatment for neonatal sepsis
Huynh et al. (BIRDY Study Group), 2021^31^	Cambodia, Madagascar, Senegal	Nine urban and rural hospitals	• 3688 neonates recruited during pregnancy, followed until day 28	2012–2018	Culture-positive incidence per 1000 live births: Cambodia, 6.5 (95% CI: 2.7–15.6); Madagascar, 15.2 (95% CI: 10.6–21.8); Senegal, 10.2 (95% CI: 4.8–21.3)	• *Klebsiella* spp. (24.4%; 11/45) • *E. coli* (22.2%; 10/45) • *Staphylococcus* spp. (24.4%; 11/45)	• 31.0% (13/42) of *Klebsiella* spp. resistant to both ampicillin and gentamicin • 48.0% (12/25) gram-negative isolates resistant to cefotaxime • 46.4% (13/28) gram-negative isolates resistant to gentamicin
Russell et al. (NeoOBS network), 2022^7^	Bangladesh, Brazil, China, Greece, India, Italy, Kenya, South Africa, Thailand, Uganda, Viet Nam	19 urban and rural hospitals	• 3204 infants < 60 days presenting to hospital with > 2 sepsis criteria, followed for 28 days	2018–2020	17.6% (564/3204)	• *K. pneumoniae* (23.4%; 132/564) • *Acinetobacter* spp. (12.8%; 72/564) •*S. aureus* (9.6%; 54/564) • *E. coli* (8.3%; 47/564)	• 57.7% (75/130 tested) of *K. pneumoniae *isolates resistant to gentamicin, 75.0% (96/128) resistant to commonly used third-generation cephalosporins and 32.6% (43/132) resistant to meropenem • 71.4% (50/70) *Acinetobacter* spp. resistant to meropenem • 35.6% (16/45) *E. coli* isolates resistant to third-generation cephalosporins

CI: confidence intervals; MDR: multidrug resistance.

Note: MDR was defined as intermediate (when a pathogen has higher minimum inhibitory concentration but is not yet resistant) or resistant to at least one drug of three of the following classes: aminoglycosides, carbapenems, cephalosporins, fluoroquinolones and piperacillin–tazobactam.

Broadly, recent data highlight the emergence of *Klebsiella* spp. and *Acinetobacter* spp., particularly carbapenem-resistant *Acinetobacter* spp., as key pathogens causing neonatal sepsis in the hospital setting. *Escherichia coli*, *Staphylococcus aureus* and *Streptococcus agalactiae* are still responsible for a significant portion of community-acquired neonatal sepsis.^7^^,^^18^^,^^29^ Genomic analyses show that extended-spectrum β-lactamases – particularly cefotaximase-Munich 15 (CTX-M) and carbapenemase-producing pathogens (mainly those harbouring *bla*_OXA-48_-like and class B metallo-β-lactamases) – are responsible for most of the excess neonatal mortality. This circumstance is because of the high rates of resistance to multiple antibiotic classes, including the empirical therapies currently recommended by WHO.^5^^,^^6^^,^^28^

### Defining priority antibiotics

The current antibiotic development pipeline relies heavily on β-lactam + β-lactamase inhibitor combinations (Table 2), many of which are ineffective against the increasingly common resistance genotypes responsible for the global burden of neonatal morbidity and mortality. Although several antibiotic candidates are under development in adults, the focus for neonates should be on developing agents likely to be effective against the pathogens and resistance mechanisms most widely prevalent in settings with the highest neonatal sepsis mortality, as outlined in Table 1.^33^

**Table 2:** Antibiotics for adult or paediatric use recently approved or in development with efficacy or the potential for efficacy against priority pathogens, 2022

Pathogen	Recently approved antibiotics for use in adults	Antibiotics in development in adult trials	Recently approved antibiotics for use in children	Antibiotics in development in paediatric trials
ESBL-producing Enterobacterales	Vaborbactam + meropenem; relebactam + imipenem + cilastatin^a^; cefiderocol; ceftolozane + tazobactam; ceftazidime + avibactam	Phase I Zidebactam + cefepime; nacubactam + cefepime; ETX-0282 + cefpodoxime; VNRX-7145 + ceftibuten; ARX-1796; xeruborbactam + QPX-2014 Phase III Sulopenem;·enmetazobactam + cefepime; cefepime + taniborbactam; aztreonam + avibactam	Ceftolozane + tazobactam; ceftazidime + avibactam (for complicated intra-abdominal infections)	Phase II Ceftazidime + avibactam (neonates and infants) Phase III Relebactam + imipenem + cilastatin^a^ (neonates and children); ceftolozane + tazobactam (neonates and children)
Carbapenemase-producing Enterobacterales	Meropenem + vaborbactam; imipenem + relebactam + cilastatin^a^; plazomicin; eravacycline;·cefiderocol; ceftazidime + avibactam^a^	Phase I Zidebactam + cefepime; nacubactam + meropenem; ETX-0282 + cefpodoxime; VNRX-7145 + ceftibuten; SPR-206; ARX-1796^a^; xeruborbactam + QPX-2014 Phase III cefepime + taniborbactam; enmetazobactam + cefepime^a^; aztreonam + avibactam	None	Phase II Ceftazidime + avibactam^a^ (neonates and infants) Phase III Relebactam + imipenem + cilastatin^a^ (neonates and children)
Carbapenem-resistant *Acinetobacter baumannii* complex	Cefiderocol	Phase I Zidebactam + cefepime; SPR-206; zifanocycline; TP-6076; xeruborbactam + QPX-2014 Phase III Durlobactam + sulbactam	None	Phase III Cefiderocol (children)
Methicillin-resistant *Staphylococcus aureus*	Omadacycline; dalbavancin; oritavancin; telavancin; contezolid (approved in China)	None	Linezolid; dalbavancin	Phase I Oritavancin, omadacycline (children) Phase II Tedizolid (neonates and children) Phase IV Telavancin
Vancomycin-resistant *Staphylococcus aureus*	None	None	None	None
Vancomycin-resistant *Enterococcus faecium*	Contezolid	Phase I Delpazolid	Linezolid	Phase III Tedizolid (neonates and children), cefiderocol (infants and children)
Carbapenem-resistant *Pseudomonas aeruginosa*	Cefiderocol	Phase I Zidebactam + cefepime; SPR-206	None	None

ESBL: extended-spectrum β-lactamase; KPC: *Klebsiella pneumoniae* carbapenemase; MBL: metallo-β-lactamase.

^a^ These antibiotics do not exhibit activity against MBL-producing pathogens.

Notes: Data obtained from publicly available reference sources.^8^^,^^9^ Enterobacterales include *Enterobacter* spp., *Escherichia coli*, *Klebsiella pneumoniae*, *Morganella* spp., *Proteus* spp., *Providencia* spp. and *Serratia* spp..

Ideally, these antibiotics should have a good safety profile, predictable pharmacokinetic parameters and adequate central nervous system penetration to enable the treatment of both neonatal sepsis and meningitis. To ensure that these antibiotics are accessible and affordable for all health-care settings, prioritized antibiotics should also have the potential for administration using short infusion times and less frequent dosing requirements, with prolonged stability at ambient temperatures. Only a limited number of agents fit these parameters.

#### Priority candidates

Using the criteria just outlined, we identified three potential priority candidates (Table 3).

**Table 3:** Potential neonatal priority antibiotics for accelerated development, 2022

Priority antibiotic in development	Priority pathogens against which activity is anticipated	Development stage and clinical indication	Other benefits	Limitations
**Cefiderocol** – a siderophore cephalosporin that forms a complex with iron which is actively transported into the bacterial cell via iron receptors	• Activity against ESBL-producing *Escherichia coli* and *Klebsiella pneumoniae* • Activity against carbapenem-resistant *Pseudomonas aeruginosa* and *Acinetobacter baumannii*, although warnings exist against its use for the latter infections in critically ill patients • Activity against gram-negative carbapenemases, including class B MBLs	• Phase III trial in adults (complicated urinary tract infection and hospital- and ventilator-acquired pneumonia in adults) – complete • Phase II trial in children > 3 months – recruiting	• Likely cerebrospinal fluid penetration • Predictable pharmacokinetic profile^34^	Requires infusions over 3 hours, three times a day
**Cefepime **+** taniborbactam** – anti-pseudomonal fourth-generation cephalosporin plus a new cyclic boronate β-lactamase inhibitor	• Activity against ESBL-producing *E. coli* and *K. pneumoniae* • Activity against gram-negative carbapenemases, including clinically relevant class B MBLs • Activity against carbapenem-resistant *P. aeruginosa*	• Phase III trial in adults – complete (complicated urinary tract infection)	• Likely cerebrospinal fluid penetration	Possible neurotoxicity effects associated with cefepime
**Sulbactam **+ **durlobactam** – dual β-lactamase inhibitor combination therapy	• Activity against MDR *A. baumannii* complex • Activity against class A, C and D β-lactamase producing Enterobacteriaceae	• Phase III trial in adults – complete	• Likely cerebrospinal fluid penetration	Requires infusions over 3 hours, four times a day

ESBL: extended-spectrum β-lactamase; MBL: metallo-β-lactamase; MDR: multidrug resistant.

##### Cefiderocol

This antibiotic is a siderophore cephalosporin that is included in two trials in children which are already recruiting (NCT04335539, NCT04215991). This antibiotic provides promise given its activity against extended-spectrum β-lactamase and metallo-β-lactamase resistance mechanisms, as well as its predictable pharmacokinetic profile and likely adequate central nervous system penetration.^35^ In 2021, cefiderocol was added to the *WHO model list of essential medicines for children* in the Reserve category, with the goal of promoting access.

##### Cefepime + taniborbactam

This antibiotic is a combination antibiotic incorporating an anti-pseudomonal cephalosporin with a new β-lactamase inhibitor. This combination also has activity against metallo-β-lactamase and extended-spectrum β-lactamase resistance mechanisms, with cefepime often retaining activity against carbapenem-resistant *Pseudomonas* spp. Pharmacokinetic data for cefepime are already published,^36^ and a paediatric investigation plan and paediatric study plan have been approved, with a development programme already underway in collaboration with the Global Antibiotic Research and Development Partnership.

##### Sulbactam + durlobactam

This antibiotic is a new dual β-lactamase inhibitor combination that is the only antibiotic currently under development (in adults) against MDR *A. baumannii*. This combination has shown promising efficacy and safety results in a recent phase III trial in adults with carbapenem-resistant *A. baumannii* complex infections.^37^ This agent also provides activity against class A, C and D β-lactamases.^38^ A paediatric investigational plan is in place and a new drug application is anticipated in 2022.

#### Other agents

While we highlight these three antibiotics as leading agents for accelerated development for neonates, other agents could potentially expand the options for management of MDR infections in children. These include other β-lactam and β-lactamase inhibitor combinations such as ceftolozane + tazobactam, meropenem + vaborbactam, ceftazidime + avibactam, imipenem + relebactam, and aztreonam + avibactam. The development status of these agents is summarized in Table 2. However, many of these options would either be less applicable to resource-constrained health settings or less effective against pathogens causing the greatest burden of neonatal morbidity and mortality, given many new β-lactam + β-lactamase inhibitor combinations have restricted activity against metallo-β-lactamase resistance mechanisms. Any list will change over time and will need to be closely aligned with the WHO Essential Medicines List for Children, particularly the Reserve antibiotics list. Alignment will also be needed with the WHO neonatal sepsis guidelines and the WHO essential medicines list antibiotic book.^39^

#### Older antibiotics

Alongside efforts focused on developing neonatal dosing and licensing for these new candidate drugs, enhanced pharmacokinetic and efficacy data for older antibiotics should also be prioritized, given the activity of agents such as fosfomycin and colistin against many neonatal priority pathogens. Fosfomycin, for example, has a broad susceptibility profile and promising safety profile,^40^ with pharmacokinetic studies recently clarifying dosing parameters for this antibiotic in neonates.^41^^,^^42^ Fosfomycin also penetrates the blood–brain barrier and has the advantage of only requiring twice-daily dosing, without the need for a prolonged infusion.^41^

### Modelling pharmacokinetic data

To improve the quality of neonatal-specific pharmacokinetic and pharmacodynamic data and to clarify dosing regimens for new and existing antibiotics more quickly than traditional pharmacokinetic studies allow, more efficient approaches can be developed.^24^ Population pharmacokinetic modelling – obtaining minimal blood samples from each participant – has become an accepted method in pharmacokinetic and pharmacodynamic trials in children. This approach allows the integration of sparse data using mathematical models to ascertain dosing regimens that can be used in efficacy trials, or to provide labelling guidance for old antibiotics.^43^

Pharmacokinetic and pharmacodynamic parameters could be promptly defined by including pharmacokinetic sampling in international trials and ensuring that parallel recruitment occurs for all stages of prematurity and postnatal age.^14^ For drug classes with predictable side-effect profiles, pharmacokinetic and pharmacodynamic trials should start as soon as sufficient adult safety and efficacy data are available, with rapid development of inclusion criteria for single-dose pharmacokinetic studies for neonatal cohorts.

### Harmonizing neonatal trials

The few neonatal antibiotic regulatory trials that have been performed are generally independent, sponsor-initiated studies, conducted with the assistance of contract research organizations. Strategic neonatal trials have been led by academic groups focused on influencing policy and prescribing guidelines. Historically, differences in the key trial elements exist between regulatory and investigator-led strategic trials. Recent collaborative efforts are gradually closing these gaps, and guidance on the principles to harmonize recruitment strategies, key inclusion and exclusion criteria, and safety reporting has been published, which enables similar benchmarks across studies.^14^ Standardization of these criteria across regulatory and strategic trials would be an efficient way to facilitate clinical trial recruitment, a strategy that has been successfully used for researching treatment options for paediatric tuberculosis and human immunodeficiency virus infection (Box 2).^44^ By building on global neonatal clinical trial networks and the work of the Clinical Trials Transformation Initiative, using a collaborative approach with standardized protocols, the recruitment into and completion of neonatal antibiotic trials could be optimized.^45^

Box 2 **Embedding neonatal drug development in strategic trials**

The time for new drugs to treat MDR pathogens to reach the market could be reduced by embedding pharmacokinetic, safety and efficacy studies in global trials in a network of well-established trial sites.

A potential achievable scenario is outlined as follows:

1. A strategic multicentre global neonatal sepsis trial is investigating new combinations of off-patent antibiotics active against MDR gram-negative organisms to treat neonatal sepsis.
2. A new agent (drug x*)* with efficacy against a neonatal priority pathogen has been developed by a pharmaceutical company and has completed single-dose pharmacokinetic trials to ascertain an appropriate paediatric dose.
3. To facilitate and expedite the phases of clinical trial development to ensure this new agent is promptly approved for neonates, further pharmacokinetic trials for drug x are embedded into a multicentre strategic trial, involving sites with experience conducting high-level pharmacokinetic trials. Validation of pharmacokinetic and pharmacodynamic parameters can be achieved in multiple sites engaged in the strategic trial.
4. Subsequently, multidose pharmacokinetic, efficacy and safety data for drug x can be obtained via a new arm within the strategic global neonatal sepsis trial.
5. Drug x is approved for use in neonates within a shorter time from adult licensing, closing the current clinical lag in neonatal drug development.
6. Enhanced population-wide active post-marketing pharmacovigilance could generate further safety data, including monitoring for the evolution of treatment resistance.

MDR: multidrug resistant.

### Enhancing trial recruitment

Collaboration within the neonatal community – including medical professionals, researchers, the pharmaceutical industry and the Global Antibiotic Research and Development Partnership – to simplify the regulatory drug development path has been advocated for many decades and neonatal consortia to address the complex challenges of drug development are emerging.^46^ The International Neonatal Consortium is gathering important data to standardize neonatal physiological variables and is developing a database of important real evidence on a variety of neonatal diseases, although not sepsis.^34^ Measures to ensure that neonatal clinical research communities work collaboratively to conduct basic science research and collate pharmacokinetic and pharmacodynamic data, alongside the coordination of study end points, could further streamline drug development.^46^

For this collaboration and coordination to occur, a shift is needed from single independent studies to global network approaches involving multiple stakeholders in public–private partnerships incorporating academia, industry and nongovernmental bodies. Where possible, standardized study designs could be developed with industry and regulator involvement. Enrolling neonates with both community- and hospital-acquired infections into these trials, with representation from both resource-rich and resource-constrained health-care settings, would help to ensure the generalizability and global applicability of findings.^14^^,^^46^ As well as establishing dosing guidance and identifying antibiotics with an improved efficacy profile to tackle the growing number of infections caused by MDR pathogens, these, potentially platform-based, trials could also explore improved diagnostic tools for neonatal sepsis and other stewardship initiatives to focus the optimal use of new agents.^47^

## Discussion

The inadequate pace of development of antibiotics for neonates threatens the attainment of the sustainable development goals.^12^ Despite the rising global burden of morbidity and mortality caused by MDR gram-negative bacteria, few clinical trials of drugs with efficacy against these pathogens are successfully enrolling neonates globally.^12^^,^^28^ The economic, regulatory and feasibility challenges inherent to the neonatal drug development process have resulted in a lack of safe and effective treatment options to manage serious bacterial infections in babies born in low-, medium- and high-income settings. To address this situation, we propose that an international, collaborative, multiexpert stakeholder group be established, which focuses on streamlining the development of the defined priority antibiotics for neonates. This collaboration could be achieved through a consensus group led by WHO working with the Global Antibiotic Research and Development Partnership together with regulators, academic clinical trial networks, potential donors and relevant industry sponsors. The group should build on prior successful and innovative multidisciplinary research methods that facilitate the management of severe infections in neonates.^48^

Examples of antibiotic development projects that have successfully initiated these strategies include the recent collaboration between the Global Antibiotic Research and Development Partnership and Penta (the NeoSep 1 trial; ISRCTN48721236) and Venatorx Pharmaceuticals (the GARDP–Venatorx collaboration). The NeoSep 1 trial is the first global hospital-based neonatal sepsis trial comparing new combinations of off-patent antibiotics to WHO-recommended antibiotics. The GARDP–Venatorx collaboration, which is supported by the National Institute of Allergy and Infectious Diseases, Wellcome Trust and Biomedical Advanced Research and Development Authority, among others, aims to accelerate the development of cefepime + taniborbactam. Such successful development partnerships – supported by industry, government and academic funding – can be used to enable the rapid development of antibiotics, and to perform safety, pharmacokinetic, pharmacodynamic and efficacy trials across a global network. Ideally, these programmes will use new study designs, such as adaptive trials, that are supported by industry and regulatory bodies, to maximize the likelihood of achieving enrolment targets.^49^ To ensure generalizable findings and to enhance research capacity in the settings where these medicines are most urgently needed, resource-constrained health settings must be included in these trials. One option might be to build on existing collaborations such as those established across Asia, South America and sub-Saharan Africa within the NeoOBS study.^7^

These promising collaborations provide examples of strategies that can reduce the currently unacceptable excess mortality caused by antimicrobial resistance in neonates. Evidence is increasing that suggests the scale of antimicrobial resistance and its burden on human health is even greater than previously appreciated; thus, global collaboration to ensure adequate research and development, capacity-building and funding directed to this health priority is vital.^2^^,^^50^ Expediting approval processes using data from strategic trials led by international clinical trial consortia has a proven track record in child health (Box 2).^44^ Such strategies require renewed attention to enhance progress against morbidity and mortality caused by MDR neonatal sepsis.

Evidence generated should be rapidly translated into updated clinical guidelines to prevent the currently unnecessary mortality as a result of antimicrobial resistance in neonatal sepsis. Concurrently, prescribing of antibiotics within appropriate stewardship frameworks is important so as to preserve their utility, as is enhanced capacity for infection, prevention and control strategies, and vaccine development strategies. Now is the time for a renewed focus to improve the funding, collaboration and research efforts urgently required to tackle the pressing threat of antimicrobial resistance to human health, particularly to the vulnerable neonatal population.
